# CSE triggers ferroptosis via SIRT4-mediated GNPAT deacetylation in the pathogenesis of COPD

**DOI:** 10.1186/s12931-023-02613-0

**Published:** 2023-12-01

**Authors:** Congping Li, Fei Chen, Liangfen Lin, Jiwei Li, Yamei Zheng, Qingyun Chen

**Affiliations:** 1 Pulmonary and Critical Care Medicine, Hainan Affiliated Hospital of Hainan Medical University, Haikou City, Hainan Province 570311 China; 2 Department of Laboratory, AffIliated to Shanghai Jiao Tong University School of Medicine Shanghai Children’s Medical Center, Hainan Branch, Sanya City, Hainan Province 572000 China; 3 Pulmonary and Critical Care Medicine, DingAn People’s Hospital, Dingan City, Hainan Province 571200 China; 4 Pulmonary and Critical Care Medicine, Hainan General Hospital (Hainan Affiliated Hospital of Hainan Medical University), No.19 Xiuhua Road, Xiuying District, Haikou City, Hainan Province 570311 China

**Keywords:** Chronic Obstructive Pulmonary Disease, Cigarette smoke extract, Ferroptosis, GNPAT, SIRT4

## Abstract

**Background:**

It is now understood that ferroptosis plays a significant role in the progression of chronic obstructive pulmonary disease (COPD) induced by cigarette smoke extract (CSE). However, the mechanisms underlying this relationship remain largely unclear.

**Methods:**

In this study, we established a COPD mouse model through exposure to cigarette smoke particulates, followed by H&E staining, analysis of bronchoalveolar lavage fluid, and immunohistochemistry assay. A549 cells were exposed to increasing concentrations of CSE, with the addition of the ferroptosis activator erastin or the inhibitor Fer-1. Cell viability, LDH (lactate dehydrogenase) release, inflammatory cytokines, total ROS (reactive oxygen species), and lipid ROS were measured using the corresponding assay kits. The acetylation level of GNPAT was determined through immunoprecipitation. We assessed the expression levels of molecules involved in plasmalogen biosynthesis (FAR1, AGPS, and GNPAT), GPX4, and SIRT4 using quantitative real-time PCR, western blot analysis, and immunofluorescence staining.

**Results:**

CSE-induced lung tissue damage was initially observed, accompanied by oxidative stress, ferroptosis, and increased plasmalogen biosynthesis molecules (FAR1, AGPS, and GNPAT). CSE also induced ferroptosis in A549 cells, resulting in reduced cell viability, GSH, and GPX4 levels, along with increased LDH, ROS, MDA (malondialdehyde) levels, oxidized lipids, and elevated FAR1, AGPS, and GNPAT expression. Knockdown of GNPAT mitigated CSE-induced ferroptosis. Furthermore, we found that CSE regulated the acetylation and protein levels of GNPAT by modulating SIRT4 expression. Importantly, the overexpression of GNPAT countered the inhibitory effects of SIRT4 on ferroptosis.

**Conclusions:**

Our study revealed GNPAT could be deacetylated by SIRT4, providing novel insights into the mechanisms underlying the relationship between CSE-induced ferroptosis and COPD.

**Supplementary Information:**

The online version contains supplementary material available at 10.1186/s12931-023-02613-0.

## Introduction

Chronic obstructive pulmonary disease (COPD) is a persistent airway condition characterized by a gradual decline in lung function, resulting in airway constriction and breathing difficulties [1]. It ranks as the third leading cause of global mortality and constitutes a widespread public health concern [2]. Cigarette smoke extract (CSE) exposure is widely recognized as the primary risk factor for COPD, primarily causing direct injury to bronchoalveolar epithelial cells, which drives the progression of the disease [3]. Traditional treatments for COPD, such as bronchodilators and glucocorticoids, primarily focus on symptom relief, highlighting the need to investigate the molecular mechanisms responsible for CSE-induced cellular damage contributing to COPD development.

More recently, ferroptosis, a newly characterized form of cell death dependent on the presence of iron, has gained significant interest. It is distinguished by the excessive accumulation of lipid peroxidation and reactive oxygen species (ROS), setting it apart from other regulated cell death mechanisms like apoptosis, necrosis, and autophagy at both the morphological and molecular levels [4, 5]. One fo the essential mediators of ferroptosis is glutathione peroxidase 4 (GPX4), which is responsible for converting lipid hydroperoxides into non-toxic lipids [6]. It is now understood that GPX4’s activity relies on glutathione (GSH) availability as a cofactor. When GPX4 is inactivated or GSH is depleted, lipid hydroperoxides accumulate, ultimately triggering ferroptosis [7, 8]. There is an increasing consensus suggesting that CSE exposure can potentially induce ferroptosis in epithelial cells, contributing to COPD pathogenesis [9–11]. However, the precise mechanism by which CSE initiates ferroptosis and damages alveolar epithelial cells remains incompletely understood.

Plasmalogens, a specific class of phospholipids enriched in polyunsaturated fatty acids, are crucial for various cellular processes, including membrane structure, signal transduction, and lipid metabolism [12]. Plasmalogen biosynthesis involves key enzymatic steps, with Fatty acyl-CoA reductase 1 (FAR1) converting fatty acids into fatty alcohols. Additionally, glyceronephosphate O-acyltransferase (GNPAT or DHAP-AT) and alkylglycerone phosphate synthase (AGPS), two peroxisomal enzymes, play pivotal roles in this process. GNPAT facilitates the acylation of dihydroxyacetone phosphate, while AGPS catalyzes the acylation of alkylglycerone phosphate [13].

Sirtuins, a family of deacetylases, comprise seven members (SIRT1-SIRT7) that display extensive conservation across various species [14]. Several proteins in the Sirtuin family have been reported to be closely associated with ferroptosis. For instance, SIRT1 can prevent ferroptosis and mitigate the harm to lung epithelial cells caused by exposure to heat stress by deacetylating the p53 protein [15]. Moreover, the resistance observed in cases of SIRT3 deficiency to autophagy-dependent ferroptosis induced by high glucose and erastin implies that targeting SIRT3 might hold promise as a therapeutic approach for the treatment of gestational diabetes mellitus (GDM) [16]. In addition, silencing SIRT6 overcomes sorafenib resistance by promoting ferroptosis via inactivating the Keap1/Nrf2 signaling pathway in gastric cancer [17]. Interestingly, a study highlighted SIRT4’s ability to protect human pulmonary microvascular endothelial cells (HPMECs) from CSE-induced stress [18]. Moreover, evidence suggests that SIRT4 can promote GNPAT degradation through deacetylation [19]. Therefore, it is highly conceivable that SIRT4-mediated GNPAT deacetylation contributes to COPD development by mediating CSE-triggered ferroptosis.

This study aimed to investigate the impact of CSE on lung tissues and human alveolar epithelial cells, emphasizing its role in inducing ferroptosis. To validate our hypothesis, a series of experiments was conducted to confirm the involvement of SIRT4-mediated GNPAT deacetylation in triggering ferroptosis upon exposure to CSE. These findings provide insights into the mechanisms of COPD pathogenesis and open up new avenues for targeted therapy for this patient population.

## Methods

### Mouse models

A total of sixteen female C57BL/6 strain mice, aged 6–8 weeks and weighing 20–25 g, were acquired from the Shanghai Animal Laboratory Center (Shanghai, China). These mice were housed in a specific-pathogen-free animal facility and provided *ad libitum* access to food and water. The mice underwent adaptive feeding for a week, after which they were assigned randomly into normal and COPD model groups with 8 mice per group. A smoking chamber (measuring 70 cm × 50 cm × 40 cm) was custom-made to establish the COPD model group, as previously described in the literature [20, 21]. The mice in COPD group were placed inside the chamber and subjected to whole-body exposure to 10 cigarette smokes (each cigarette contains 1.2 mg of nicotine and 10 mg of tar) for 2 h once a day for six months. Following the final exposure, the mice were euthanized under anesthesia, and a ligation was performed on the trachea and one of the lung lobes. A puncture needle was then inserted into the upper part of the trachea. After unilateral lung perfusion (3–5 times) using 0.3 ml of PBS, the bronchoalveolar lavage fluid was obtained and stored temporarily at 4 °C. Lung tissues that were not irrigated were preserved using a combination of partial freezing at -80 °C and fixation with neutral-buffered formalin overnight.

### Biological analysis of lavage solution

A sterile container was used to collect bronchoalveolar lavage fluid to count neutrophils, macrophages, and lymphocytes. The collected cells were stained using the May-Grunwald Giemsa method and analyzed through standard microscopy. The resulting cell counts were recorded.

### Histology and immunohistochemistry

The lung tissues obtained from COPD and normal mice were subjected to ethanol dehydration, followed by embedding in paraffin. Subsequently, the tissues were sliced into sections measuring 5 μm in thickness. Following the process of deparaffinization and rehydration, specific sections were subjected to staining with hematoxylin (Solarbio, H8070, Beijing, China) and eosin (Sangon, A600190, Shanghai, China) for histological evaluation. Then, the alveolar wall thickness and mean alveolar septa under HE staining were measured by ImageJ software. For immunohistochemistry, selected sections of lung tissues from COPD and normal mice were deparaffinized, rehydrated, and placed in sodium citrate buffer to retrieve antigens. Subsequently, the sections were blocked with serum and subjected to incubation with primary antibodies targeting Bax (ab32503; Abcam), GPX4 (ab219592; Abcam) and GNPAT (14931-1-AP, Proteintech), which were diluted in PBS at 4 °C overnight. Afterward, the sections were incubated with an HRP-conjugated secondary antibody for 1 h at room temperature and visualized using a microscope imaging system (Nikon, Tokyo, Japan).

### CSE preparation

To generate a solution of CSE for this study, a single unfiltered cigarette containing 1.2 mg of nicotine and 10 mg of tar was lit, and its smoke was continuously drawn into a sterile 10mL solution of phosphate-buffered saline (PBS) using a negative pressure suction device, resulting in a solution known as CSE. This CSE solution was purified through a 0.22 μm filter to remove any remaining cigarette residue and bacteria. Upon completion, the liquid was characterized as a CSE solution with a purity of 100%. CSE solution was diluted with DMEM medium to 0.1%, 0.5%, 2%, and 5% CSE concentration and used in subsequent experiments within 15 min after preparation.

### Cell culture and intervention

The human type II alveolar epithelial cell line A549 was obtained from Procell Life Science and Technology Co., Ltd (Wuhan, China) and cultured in DMEM medium (Gibco) supplemented with 10% FBS (Gibco), penicillin (100 U/ml), and streptomycin (100 U/ml) at 37 °C with 5% CO_2_. According to the different experimental requirements, A549 cells were classified into different groups as follows: (1) Blank group (without treatment), 0.1%, 0.5%, 2% and 5% CSE groups; (2) Blank group, 5% CSE group, 5% CSE + DMSO group, 5% CSE + Erastin group that cells were co-treated with 5% CSE and 2.5 mM erastin (Selleck Chemicals, USA) and 5% CSE + Fer-1 group that cells were co-intervened with 5% CSE and 1 mM Fer-1 (Med Chem Express, USA); (3) A549 cells were transfected with sh-negative control (sh-NC) and sh-GNPAT (Ruibio Biotech Co., Ltd., Beijing, China) for 48 h, followed by 5% CSE treatment and divided into corresponding two groups; (4) A549 cells were transfected with overexpression plasmid SIRT4 alone or GNPAT together (GenePharma Co. Ltd., Shanghai, China), followed by 5% CSE treatment. Four groups were obtained, including blank + vector, 5% CSE + vector, 5% CSE + SIRT4, and 5% CSE + SIRT4 + GNPAT. All cell transfections were carried out using Lipofectamine 3000 as per the manufacturer’s instructions.

### CCK-8 assay

Cell viability was determined using a Cell Counting Kit-8 (CCK-8) kit (CK04, Dojindo Laboratories, Japan). In brief, A549 cells from various groups (2 × 103 cells/well) were plated in 96-well plates and incubated at 37 °C with 5% CO_2_ for 6, 12, 24, and 36 h, respectively. Subsequently, the cells in each well were treated with a complete medium containing 10 µl of CCK-8 solution and incubated for an additional 2 h. The optical density of each well was then determined at 450 nm using a microplate reader.

### LDH activity assay

The supernatants (100 µL) from different groups were collected, and lactate dehydrogenase (LDH) release was measured using an LDH Cytotoxicity Assay kit (C0016, Beyotime Institute of Biotechnology) according to the manufacturer’s instructions.

### Measurement of reactive oxygen species (ROS)

The level of total ROS was determined using a 2’,7’-dichlorofluorescein diacetate (DCFH-DA) kit (Beyotime, Shanghai, China). Cells from different groups (5 × 10^5^ cells/well) were placed into six-well plates and incubated with 10 µmol/L DCFH-DA diluted with serum-free medium for 20 min at 37 °C. After washing thrice with a serum-free medium, ROS levels were analyzed by flow cytometry.

### Lipid oxidation detection

The level of lipid oxidation was detected using the C11-BODIPY (581/591) probe (Servicebio Technology, Wuhan, China), where non-oxidized lipids were stained in red and oxidized lipids in green. Briefly, cells from different groups were washed twice with PBS and incubated with 10 µmol/L BODIPYTM 581/591 C11 at 37 °C in the dark for 30 min. The fluorescence intensity was observed under an inverted fluorescence microscope (×500 magnification).

### Enzyme-linked immunoassay (ELISA) assay

In short, the study involved collecting bronchoalveolar lavage fluid and cell supernatant in PBS. The levels of GPX4, IL-33, and IL-1α in both the bronchoalveolar lavage fluid and cell supernatant were measured using ELISA kits from Shanghai Enzyme-linked Biotechnology Co., Ltd (ml057982/ ml060706, ml063153/ml063084, ml002273/ml058010), following the manufacturer’s instructions.

### Detection of MDA and GSH content

The MDA and GSH concentrations in lung tissue samples dissolved in extraction buffer or cellular supernatant were determined using kits from Beyotime (S0131S, S0053, Shanghai, China), following the manufacturer’s instructions. MDA and GSH levels were measured individually using a microplate reader at 450 and 405 nm, respectively.

### Flow cytometry

The apoptotic rate of A549 cells was measured using an Annexin V-FITC/PI detection kit (CA1020, Solarbio, Beijing, China). Briefly, A549 cells from different groups were harvested after trypsin digestion and fixed with 70% ethanol. Then, cells were stained with Annexin V-FITC and PI in the presence of 50 mg/mL RNase A for 1 h at room temperature in darkness, which were subsequently assessed with flow cytometry.

### Transmission electron microscopy

A549 cells from various groups underwent fixation with 1% osmic acid at room temperature in the dark for 2 h, dehydration using acetone, polymerization at 60 °C for 48 h, and slicing into 60–80 nm ultrathin sections. These sections were then stained with a 2% saturated uranyl acetate alcohol solution and a 2.6% lead citrate solution and observed for any changes in mitochondrial morphology under a transmission electron microscope (×50 000 magnification, Hitachi, Tokyo, Japan).

### Immunofluorescence staining

The A549 cells from different groups were subjected to three washes with PBS, then fixation with 4% paraformaldehyde for 30 min. Subsequently, the cells were treated with 1% Triton X-100 for 20 min. Following three washes with PBS, the cells were blocked with 5% BSA for 1 h at 37 °C. Subsequently, they were incubated overnight at 4 °C with the primary antibody against GNPAT (14931-1-AP, Proteintech). Afterward, a fluorescent secondary antibody (1:300) was added and incubated for 2 h at 37 °C. Finally, the cells were stained with DAPI (1:4000) for 5 min before observation under a fluorescence microscope (×500 magnification).

### Quantitative real-time PCR

Total RNA was extracted from lung tissues or cells using Trizol reagent from Invitrogen, USA. The extracted RNA was then reverse-transcribed into cDNA using the PrimeScript™ RT reagent kit by Takara. The PCR amplification was carried out using the SYBR Green PCR kit by Takara on the ABI PRISM Step-One Real-time PCR System, manufactured by Applied Biosystems, Carlsbad, CA, USA. The relative expression levels were determined by the 2^−ΔΔCT^ method. Table 1 contains the PCR primer sequences used in the experiment.

**Table 1:** Primer pairs used for quantitative RT-PCR analysis

Gene ID	Sequence (5’- 3’)
H-GAPDH F	TGTTCGTCATGGGTGTGAAC
H-GAPDH R	ATGGCATGGACTGTGGTCAT
H-FAR-1 F	CTCACCCAACCTAAACTGGCT
H-FAR-1R	GCTTGCGATTACAGTAGGCATA
H-AGPS F	TGAGTACCAATGAGTGCAAAGC
H-AGPS R	GGTAAACCCATGCCACTAAGAG
H-GNPAT F	AAGAGAGGAGGCATGTCAGT
H-GNPAT R	AACCGAATGGCTCCAAGACG
H-SIRT4 F	TCGTAGGCTGGCCTCAATTC
H-SIRT4 R	CCCACAATCCAAGCACAGGA
M-β-actin F	CATTGCTGACAGGATGCAGA
M-β-actin R	CTGCTGGAAGGTGGACAGTGA
M-FAR-1 F	TAGTGGTCAACACGAGCCTTG
M-FAR-1 R	GGCTTACAGCAATCCAGTAATGA
M-AGPS F	AATGGATGGGGCTACAATGATTC
M-AGPS R	TCCAGACTTACTCCAAGGGTG
M-GNPAT F	GGAGCCATTCGGTTCTTTGC
M-GNPAT R	AAGTCCATTCCTGCTGCGAT

H: human; M: mouse; F: forward primer; R: reverse primer; RT: reverse transcription primer

### Western blot analysis

Total protein was isolated from lung tissues or cells using RIPS lysis buffer (Beyotime, Shanghai, China), and corresponding protein concentration was determined with BCA protein assay (Beyotime). After being mixed with loading buffer and boiled in boiling water, 30 µg proteins were separated by SDS-polyacrylamide gel electrophoresis and transferred to PVDF membranes (Millipore). After being blocked with 5% non-fat milk for 2 h, the membranes were incubated with primary antibodies against GPX4 (ab219592), FAR-1 (ab202298), AGPS (ab236621), GNPAT (ab75060) and β-actin (ab8227) from Abcam overnight at 4 °C. Afterwards, the membranes were incubated with horseradish peroxidase-linked secondary antibody (1:5000) for 2 h at room temperature. Protein bands were visualized with enhanced chemiluminescence reagent (Thermo Fisher Scientific).

### Immunoprecipitation assay

To perform the immunoprecipitation assay, we first lysed the cells using a lysis buffer. Next, the cell lysates were incubated with a cross-linked resin and 10 µg of GNPAT antibody (ab75060, Abcam) overnight at 4 °C. We eluted the protein complexes bound to the antibody the following day using an elution buffer. These eluted complexes were then subjected to a western blot assay using an acetyl-lysine antibody (ab190479, Abcam).

### Statistical analysis

GraphPad Prism 8 software (San Diego, CA, USA) was utilized for data analysis. The results were presented as mean ± standard deviation (SD). Statistical comparisons were conducted using two-sided, unpaired Student’s t-test or one-way analysis of variance (ANOVA), followed by Dunnett’s test or Tukey’s post-hoc test as appropriate. A *p*-value less than 0.05 was considered statistically significant.

## Results

### Investigation of the association between plasmalogen biosynthesis enzymes and ferroptosis in COPD mice

**Fig. 1:**
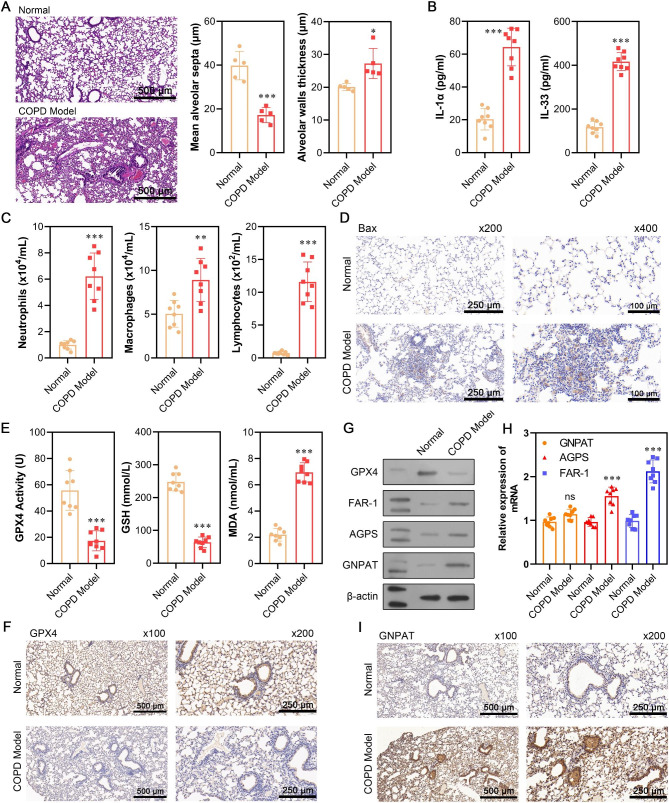
Investigation of the association between the expression of plasmalogen biosynthesis and ferroptosis in COPD mice. (**A**) H&E staining of sections of mouse lung tissues from COPD model and normal group. (**B**) The concentration of IL-33 and IL-1α in the bronchoalveolar lavage fluid was determined by ELISA assay. (**C**) The neutrophils, macrophages, and lymphocytes in bronchoalveolar lavage fluid were quantified and compared between COPD and normal groups. (**D**) Tissue damage of lung were elevated by immunohistochemistry staingin of Bax. (**E**) GPX4 activity and the contents of GSH and MDA were analyzed between COPD and normal groups. (**F**) GPX4 expression in the lung tissues of mice was tested by immunohistochemistry (×100 and ×200). (**G**) The protein expression of GPX4, FAR-1, AGPS, and GNPAT was detected via western blot analysis. (**H**) The mRNA expression of FAR-1, AGPS, and GNPAT was measured via quantitative real-time PCR. (**I**) GNPAT expression in the lung tissues of mice was tested by immunohistochemistry (×100 and ×200). Data are presented as the mean ± SD of three replicates and analyzed using Student’s t-test. ^ns^*p* > 0.05, **p < 0.01, ***p < 0.001, compared with normal. The gels were cropped reasonably

First, a COPD mouse model was established to explore the potential involvement of plasmalogen biosynthesis molecules (FAR1, AGPS, and GNPAT) in ferroptosis. H&E staining revealed extensive lung tissue damage in the COPD model group, characterized by thickened alveolar walls, reduced alveolar septa size, partial tracheal obstruction, and proliferation of goblet epithelial cells. In contrast, the normal control group displayed well-defined alveolar structures with a uniform arrangement (Fig. 1A). ELISA assay results indicated a significant rise in pro-inflammatory cytokines (IL-33 and IL-1α) in the COPD model group compared to the normal group (Fig. 1B).Additionally, bronchoalveolar lavage fluid analysis showed a significant increase in three types of inflammatory cells, including neutrophils, lymphocytes, and macrophages, in the COPD model compared to the normal group (Fig. 1C). The immunohistochemistry staining for Bax also confirmed the damage of lung tissue in COPD mice (Fig. 1D). Regarding ferroptosis markers, GPX4 and GSH levels were substantially reduced, while MDA was elevated in the COPD model group compared to the normal group (Fig. 1E). Immunohistochemistry and western blot analysis corroborated the downregulation of GPX4 in lung tissues from the COPD model (Fig. 1F and G). Furthermore, western blot analysis (Fig. 1G) and quantitative real-time PCR (Fig. 1H) confirmed increased expression of FAR-1 and AGPS at protein and mRNA level in the COPD group, while GNPAT expression only increased at protein level but not at mRNA level. These findings suggest the potential involvement of FAR-1, AGPS, and GNPAT in ferroptosis in the lungs of COPD patients. Immunohistochemical staining further supported higher expression of GNPAT in the COPD model, indicating a role for post-transcriptional or protein-level regulation in GNPAT expression (Fig. 1).

### Induction of ferroptosis by CSE and promotion of plasmalogen biosynthesis enzyme expression in human alveolar epithelial cells

**Fig. 2:**
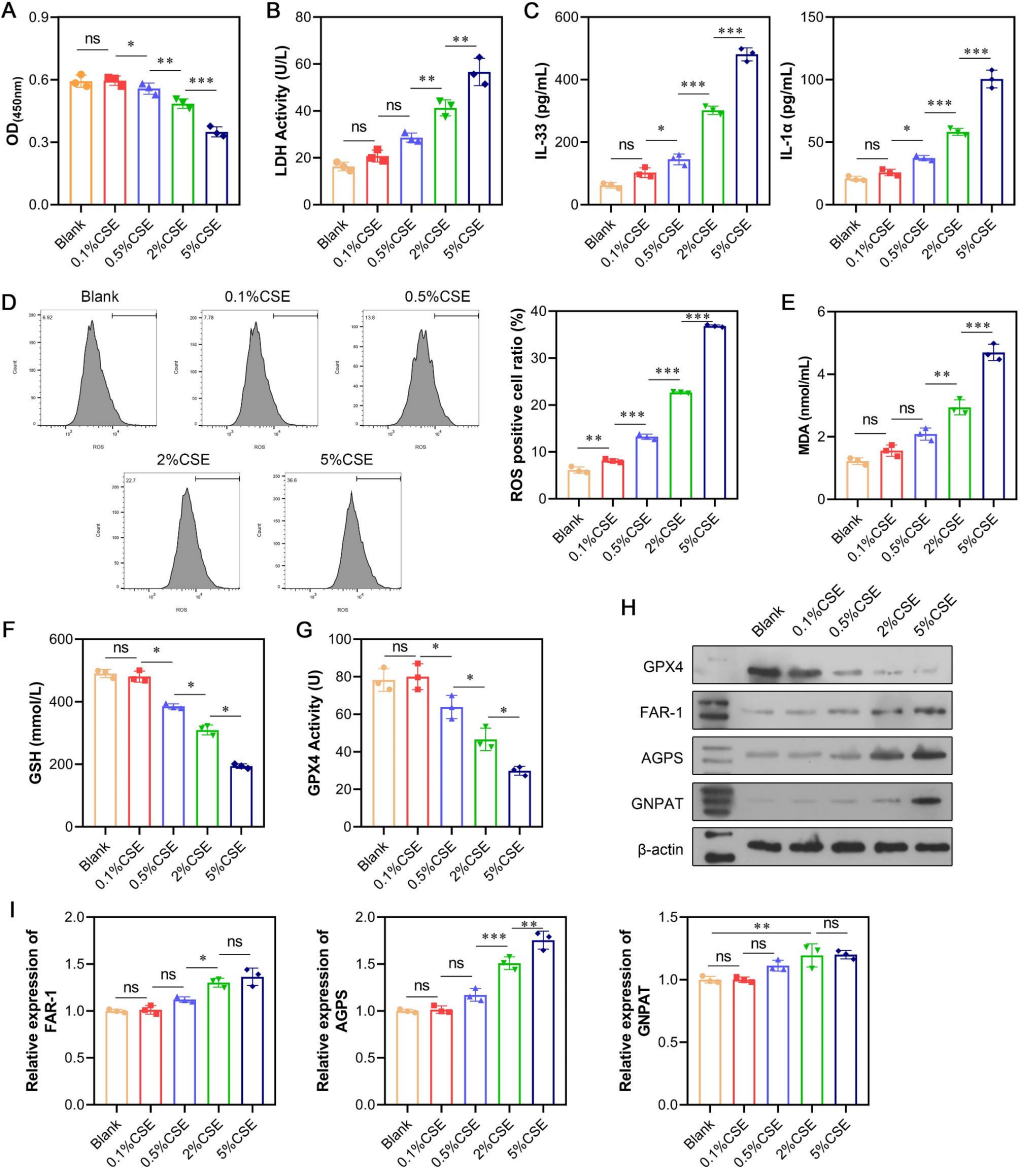
CSE triggered ferroptosis and upregulated the expression of plasmalogen biosynthesis in human alveolar epithelial cells. Human type II alveolar epithelial A549 cells were exposed to 0.1%, 0.5%, 2%, and 5% CSE for 24 h. (**A**) Cell viability was detected by CCK-8 assay. (**B**) LDH release was detected by LDH activity assays. (**C**) The concentration of IL-33 and IL-1α in the cellular supernatant was determined by ELISA assay. (**D**) The levels of total ROS were determined using a 2’, 7’-dichlorofluorescein diacetate (DCFH-DA) kit. (**E-G**) Quantification of the ferroptosis-related markers MDA, GSH, and GPX4. (**H**) The protein levels of GPX4, FAR-1, AGPS, and GNPAT were detected by western blot analysis. (**I**) The mRNA expression of FAR-1, AGPS, and GNPAT was measured via quantitative real-time PCR. Data are presented as the mean ± SD of three replicates and analyzed using one-way analysis of variance (ANOVA), followed by Dunnett’s test. ^ns^*p* > 0.05, *p < 0.05, **p < 0.01, ***p < 0.001, compared with blank. The gels were cropped reasonably

Human alveolar epithelial A549 cells were exposed to various concentrations of CSE (0.1%, 0.5%, 2%, and 5%) for 24 h. The CCK-8 assay demonstrated reduced cell viability with increasing CSE concentrations (Fig. 2A). The LDH assay revealed increased LDH enzyme activity with higher CSE concentrations, indicating cellular damage (Fig. 2B). The inflammatory cytokines IL-33 and IL-1α exhibited a dose-dependent increase in response to increased CSE concentrations, indicating heightened cellular inflammation (Fig. 2C). Flow cytometry showed a dose-dependent increase in ROS production following CSE exposure (Fig. 2D). Similarly, CSE exposure led to a significant increase in malondialdehyde (MDA) production (Fig. 2E) and a decrease in glutathione (GSH) and GPX4 levels (Fig. 2F and G). Western blot analysis revealed downregulated GPX4 and upregulated acetal phospholipid-related enzymes (FAR1, AGPS, and GNPAT) in a dose-dependent manner after CSE exposure (Fig. 2H). PCR analysis showed elevated mRNA levels of the three plasmalogen biosynthesis enzymes in a dose-dependent manner, with AGPS showing the most substantial increase and GNPAT exhibiting the least pronounced increase (Fig. 2I). Notably, while the PCR assay indicated a minor increase in GNPAT mRNA levels, the corresponding protein level showed a significant increase, suggesting a potential post-transcriptional or protein-level regulation mechanism similar to the in vivo results. Based on the substantial impact of the 5% CSE treatment, this concentration was chosen for subsequent experiments to investigate the role of GNPAT.

### Experimental validation of CSE-induced ferroptosis in human alveolar epithelial cells

**Fig. 3:**
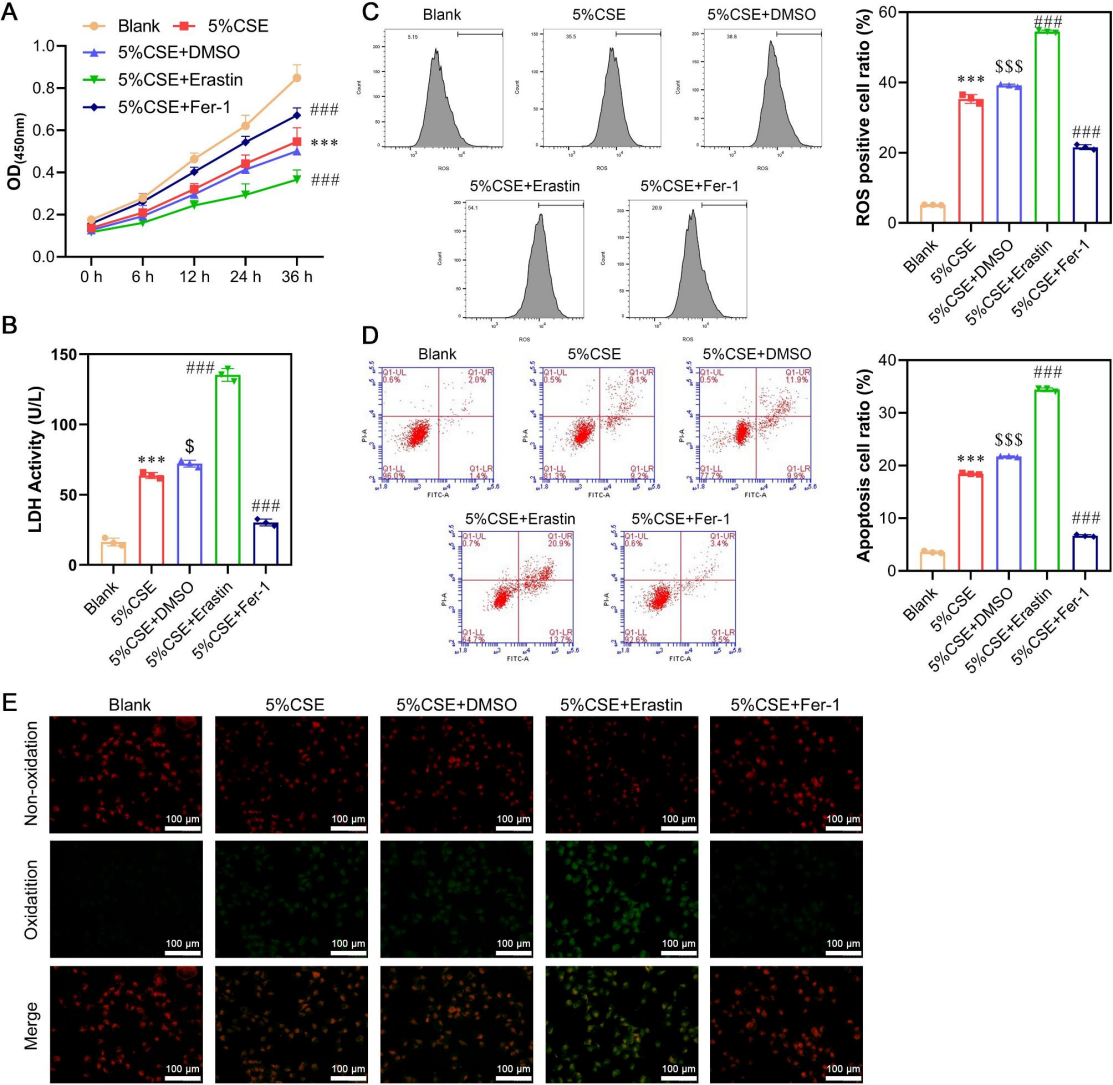
Experimental validation of CSE-induced cell damage, inflammation and ROS in human alveolar epithelial cells. A549 cells were treated with DMSO, erastin (ferroptosis activator) or Fer-1 (ferroptosis inhibitor), accompanied by 5% CSE exposure. (**A**) Cell viability was detected by CCK-8 assay. (**B**) The LDH release was detected by LDH activity assays. (**C**) The levels of total ROS were determined using a 2’, 7’-dichlorofluorescein diacetate (DCFH-DA) kit. (**D**) Cell apoptosis was determined by flow cytometry assay. (**E**) Detection of lipid oxidation by the BODIPY 581/591 C11 probe method. Data are presented as the mean ± SD of three replicates and analyzed using ANOVA, followed by Tukey’s post-hoc test. ***p < 0.001, compared with blank; $*p* < 0.05, $$$*p* < 0.001, compared with 5% CSE; ###*p* < 0.001, compared with 5% CSE + DMSO

**Fig. 4:**
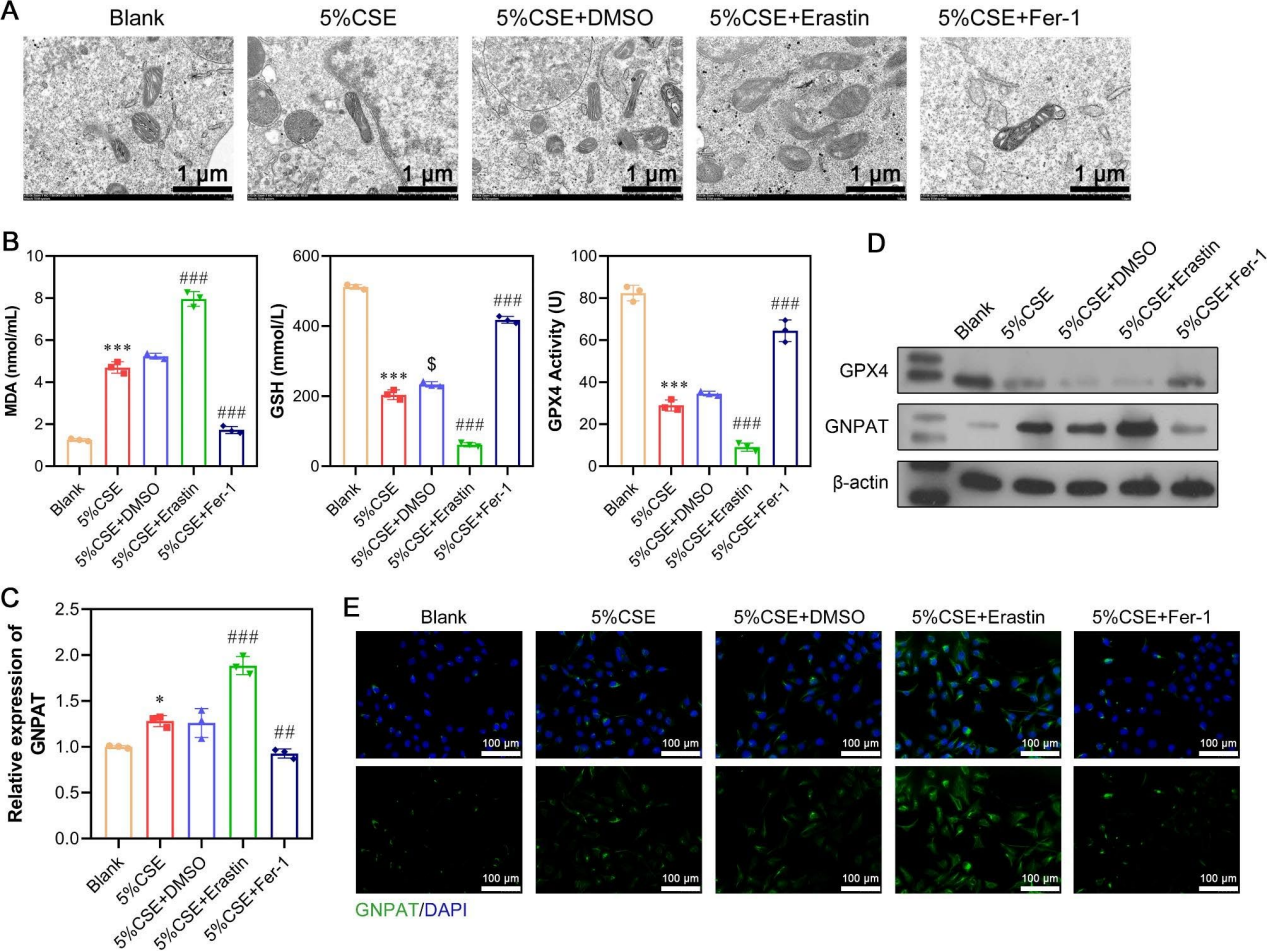
Experimental validation of CSE-induced markers associated with ferroptosis and plasmalogen biosynthesis. (**A**) Mitochondrial morphology associated with ferroptosis was determined by transmission electron microscopy. (**B**) Content detection of the ferroptosis-related markers MDA, GSH, and GPX4 by ELISA. (**C**) The mRNA expression of GNPAT was measured via quantitative real-time PCR. (**D**) The protein levels of GPX4 and GNPAT were detected by western blot analysis. (**E**) Immunofluorescence staining of GNPAT was displayed in different groups. Data are presented as the mean ± SD of three replicates and analyzed using ANOVA, followed by Tukey’s post-hoc test. *p < 0.05, ***p < 0.001, compared with blank; $*p* < 0.05, compared with 5% CSE; ##*p* < 0.01, ###*p* < 0.001, compared with 5% CSE + DMSO. The gels were cropped reasonably

To validate that CSE induced cell damage via ferroptosis, A549 cells were treated with DMSO, erastin (ferroptosis activator), or Fer-1 (ferroptosis inhibitor) in conjunction with 5% CSE exposure. The addition of DMSO to 5% CSE did not significantly affect cell viability or LDH activity compared to the group treated with 5% CSE alone, as evidenced by CCK-8 (Fig. 3A) and LDH (Fig. 3B) analyses. However, the group treated with both 5% CSE and erastin displayed decreased cell viability and increased LDH activity, indicating that erastin exacerbated the detrimental effects induced by CSE. Conversely, the group treated with 5% CSE and Fer-1 showed improved cell viability and reduced LDH activity, suggesting that Fer-1 alleviated cellular damage caused by CSE. Flow cytometry revealed that 5% CSE exposure induced ROS production and apoptosis, which were attenuated by Fer-1 and intensified by erastin (Fig. 3C and D). Analysis of lipid oxidation indicated increased lipid peroxidation in the 5% CSE + erastin group and decreased lipid peroxidation in the 5% CSE + Fer-1 group compared to the 5% CSE group (Fig. 3E). Transmission electron microscopy (TEM) analysis demonstrated that the addition of Fer-1 restored the number of mitochondrial inner ridges, whereas the supplementation of erastin further decreased the number of inner ridges (Fig. 4A). At the molecular level, exposure to 5% CSE led to increased MDA levels and decreased GSH and GPX4 levels. Western blot analysis, quantitative real-time PCR, and immunofluorescence staining revealed that erastin increased GNPAT mRNA and protein levels while decreasing GPX4 protein expression. In contrast, Fer-1 yielded the opposite effects, decreasing GNPAT and increasing GPX4 levels under the same experimental conditions (Fig. 4C and D, and 4E). These results collectively suggested that ferroptosis played a significant role in CSE-induced cell damage.

### Interference with GNPAT can alleviate the CSE-induced ferroptosis of human alveolar epithelial cells

**Fig. 5:**
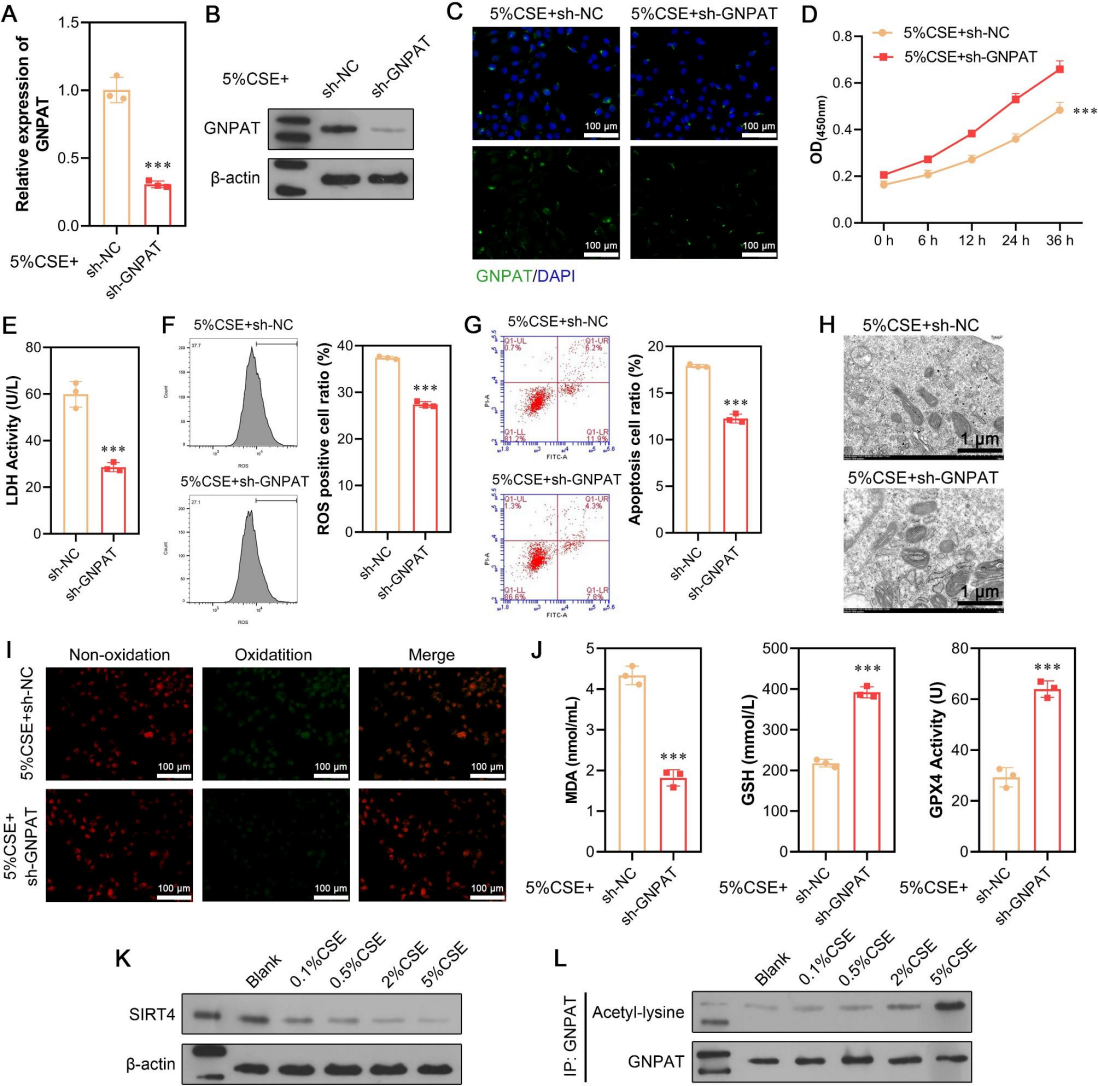
Interference with GNPAT can alleviate CSE-induced ferroptosis in human alveolar epithelial cells. A549 cells were transfected with sh-GNPAT or sh-NC for 48 h, followed by 5% CSE treatment. (**A-B**) The expression levels of GNPAT mRNA and protein were determined in the above A549 cells. (**C**) Immunofluorescence staining of GNPAT was shown. (**D**) Cell viability was detected by CCK-8 assay. (**E**) The LDH release was detected by LDH activity assays. (**F**) The levels of total ROS were determined using a 2’, 7’-dichlorofluorescein diacetate (DCFH-DA) kit. (**G**) Cell apoptosis was determined by flow cytometry assay. (**H**) Mitochondrial morphology associated with ferroptosis was determined by transmission electron microscopy. (**I**) Detection of lipid oxidation by the BODIPY 581/591 C11 probe method. (**J**) Content detection of the ferroptosis-related markers MDA, GSH, and GPX4 by ELISA. A549 cells were exposed to 0.1%, 0.5%, 2%, and 5% CSE for 24 h, followed by quantification of SIRT4 expression via western blot analysis (**K**) and GNPAT acetylation level by immunoprecipitation (**L**). Data are presented as the mean ± SD of three replicates and analyzed using Student’s t-test. ***p < 0.001, compared with sh-NC. The gels were cropped reasonably

In light of the pronounced elevation of GNPAT protein levels in CSE-induced ferroptosis, a loss-of-function assay was conducted to explore the functional role of GNPAT in vitro. First, quantitative real-time PCR (Fig. 5A), western blot analysis (Fig. 5B), and immunofluorescence staining (Fig. 5C) confirmed the successful silencing of GNPAT in CSE-treated A549 cells following sh-GNPAT transfection. Subsequent functional experiments revealed that GNPAT knockdown significantly alleviated CSE-induced ferroptosis, as evidenced by restored cell viability (Fig. 5D), decreased LDH activity (Fig. 5E), reduced ROS production (Fig. 5F), lower cell apoptosis rates (Fig. 5G), restoration of the number of mitochondrial inner ridges (Fig. 5H), and diminished lipid peroxidation (Fig. 5I). Furthermore, GNPAT inhibition resulted in increased levels of GPX4 and GSH, along with decreased levels of MDA (Fig. 5J), highlighting the suppressive effects of GNPAT knockdown on ferroptosis. Given that SIRT4 has been reported to deacetylate and downregulate GNPAT, we proceeded to examine the expression of SIRT4 and the acetylation of GNPAT in A549 cells exposed to various concentrations of CSE. Our findings revealed a significant reduction in the expression level of SIRT4 with increasing CSE concentration (Fig. 5K). In addition, we observed an increase in the acetylation level of GNPAT with rising CSE concentration, in contrast to the changes observed in SIRT4 expression (Fig. 5L). These findings suggest that CSE may regulate the acetylation and protein levels of GNPAT through the modulation of SIRT4 expression.

### Experimental validation of CSE triggering ferroptosis via SIRT4-mediated GNPAT deacetylation

**Fig. 6:**
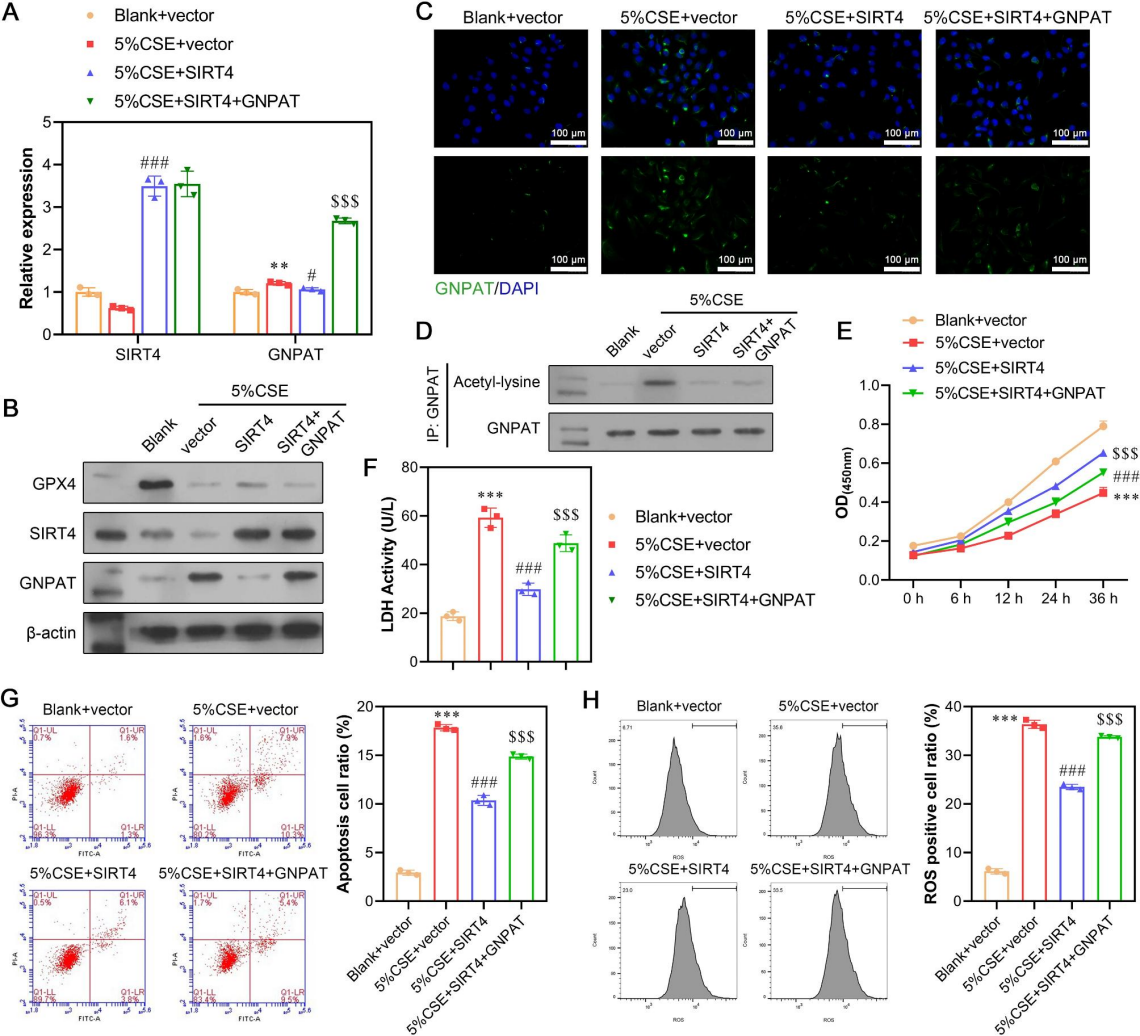
Experimental validation of CSE-induced apoptosis and ROS generation via SIRT4-mediated GNPAT acetylation. A549 cells were classified into four groups, including blank + vector, 5% CSE + vector, 5% CSE + SIRT4 and 5% CSE + SIRT4 + GNPAT. (**A-B**) The expression levels of SIRT4 and GNPAT at mRNA and protein were determined in the above A549 cells. (**C**) Immunofluorescence staining of GNPAT was shown. (**D**) GNPAT acetylation level was detected by immunoprecipitation assay. (**E**) Cell viability was detected by CCK-8 assay. (**F**) The LDH release was detected by LDH activity assays. (**G**) Cell apoptosis was determined by flow cytometry assay. (**H**) The levels of total ROS were determined using a 2’, 7’-dichlorofluorescein diacetate (DCFH-DA) kit. Data are presented as the mean ± SD of three replicates and analyzed using ANOVA, followed by Tukey’s post-hoc test. **p < 0.01, ***p < 0.001, compared with blank + vector; #*p* < 0.05, ###*p* < 0.001, compared with 5% CSE + vector; $$$*p* < 0.001, compared with 5% CSE + SIRT4. The gels were cropped reasonably

**Fig. 7:**
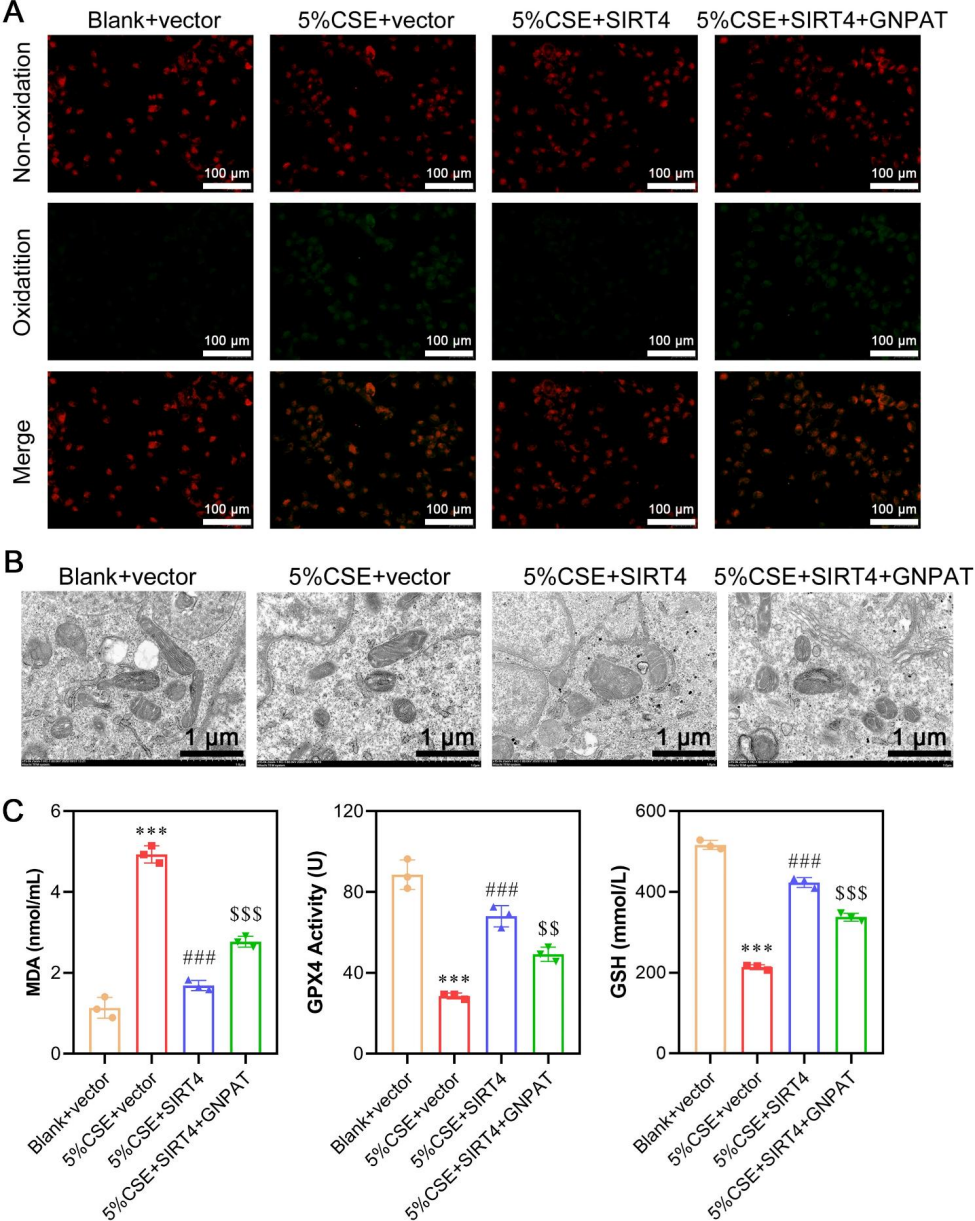
Experimental validation of CSE-induced ferroptosis-related markers via SIRT4-mediated GNPAT acetylation. (**A**) Detection of lipid oxidation by the BODIPY 581/591 C11 probe method. (**B**) Mitochondrial morphology associated with ferroptosis was determined by transmission electron microscopy. (**C**) Content detection of the ferroptosis-related markers MDA, GSH, and GPX4 by ELISA. Data are presented as the mean ± SD of three replicates and analyzed using ANOVA, followed by Tukey’s post-hoc test. ***p < 0.001, compared with blank + vector; ###*p* < 0.001, compared with 5% CSE + vector; $$*p* < 0.01, $$$*p* < 0.001, compared with 5% CSE + SIRT4

Next, we conducted rescue experiments to investigate whether SIRT4-mediated GNPAT deacetylation played a role in CSE-triggered ferroptosis. The following groups were established: blank + vector, 5% CSE + vector, 5% CSE + SIRT4, and 5% CSE + SIRT4 + GNPAT. In the presence of CSE, GNPAT mRNA levels slightly increased, while protein levels significantly increased. However, upon transfection with an overexpressed SIRT4 plasmid, GNPAT protein levels decreased considerably, suggesting that CSE and SIRT4 primarily regulate GNPAT at the protein level. The successful overexpression of GNPAT was confirmed by a substantial increase in both mRNA and protein levels following transfection. Moreover, the addition of CSE reduced both the mRNA and protein levels of SIRT4. However, transfection with an overexpressed SIRT4 plasmid significantly elevated the mRNA and protein levels of SIRT4, confirming the efficiency of the transfection procedure (Fig. 6A-B). Immunofluorescence staining of GNPAT also corroborated these results (Fig. 6C). Immunoprecipitation assays revealed that the acetylation level of GNPAT increased with the addition of CSE but decreased upon overexpression of SIRT4 (Fig. 6D). Subsequent functional assays demonstrated that GNPAT overexpression significantly reversed the suppressive effects of SIRT4 overexpression on CSE-triggered ferroptosis in A549 cells, as indicated by the CCK-8 assay (Fig. 6E), LDH assay (Fig. 6F), flow cytometry (Fig. 6G), DCFH-DA kit (Fig. 6H), BODIPY 581/591 C11 probe (Fig. 7A), TEM analysis (Fig. 7B), as well as analysis of MDA, GPX4 and GSH (Fig. 7C) activities.

## Discussion

While the involvement of ferroptosis in the pathogenesis of COPD induced by cigarette smoke is well established, the mechanisms underlying this association remain largely elusive. In this study, we observed elevated expression of plasmalogen biosynthesis molecules (GNPAT, AGPS, and FAR-1) in a COPD mouse model and CSE-exposed A549 cells accompanied by ferroptosis. Notably, plasmalogens are a class of ether phospholipids found in various human tissues, including the lungs and lung surfactants, known for their remarkable antioxidant properties [22]. Lee et al. [23] reported that genetic ablation of genes involved in alkyl-ether plasmalogen biosynthesis (*FAR1, GNPAT, AGPS*, and *AGPAT3*) leads to ferroptosis resistance. This study implies a possible relationship between plasmalogen biosynthesis enzymes (GNPAT, AGPS, and FAR-1) and CSE-induced ferroptosis.

To explore how plasmalogen biosynthesis enzymes regulate ferroptosis induced by CSE, we initially confirmed the occurrence of CSE-induced ferroptosis in human alveolar epithelial cells using the ferroptosis activator erastin and the inhibitor Fer-1. Previous research has indicated that CSE results in airway inflammation and the death of alveolar epithelial cells [24]. Our study utilized the A549 cell line, derived from human lung adenocarcinoma cells, which exhibit key characteristics of type II alveolar epithelial cells. This cell line has become a popular choice for studying COPD at the cellular level due to increasing evidence supporting its similarity to actual type II alveolar epithelial cells [25–27]. In a loss-of-function assay, we knocked down GNPAT and found that inhibiting GNPAT expression effectively mitigated CSE-induced ferroptosis. While a direct causal relationship between GNPAT and ferroptosis remains unclear, Xu et al. [28] conducted a study demonstrating that knocking down GNPAT using shRNA significantly mitigated the effects of clustered regularly interspaced short palindromic repeats-catalase by reducing the levels of plasmalogens and ROS. Moreover, GNPAT significantly enhanced the growth-suppressing effects of CDDO-Me (an antitumor agent) and induced apoptosis in melanoma cells [29]. These results suggest that regulating the generation of plasmalogens through the enzyme GNPAT plays a significant role in determining the vulnerability of cells to ferroptosis, highlighting the potential of targeting GNPAT as a therapeutic approach for diseases marked by disrupted lipid metabolism and oxidative stress.

There is mechanistic evidence suggesting that the deacetylation process catalyzed by SIRT4 may enhance GNPAT degradation [19]. In this study, we observed that with an increase in CSE concentration, the acetylation level of GNPAT increased, while the expression level of SIRT4 decreased. Subsequently, we conducted rescue experiments to explore whether the deacetylation of GNPAT by SIRT4 played a role in CSE-induced ferroptosis. As expected, the overexpression of GNPAT significantly countered the inhibitory effects of SIRT4 on CSE-induced ferroptosis. SIRT4, primarily found in mitochondria, is crucial in various metabolic processes within this organelle [30]. Its impact on ATP levels and lipid metabolism suggests that it may be involved in several mitochondrial dysfunction-related disorders and diseases [31, 32]. Additionally, SIRT4 has been shown to promote cardio-protection by increasing cellular resistance to H_2_O_2_-induced apoptosis in H9c2 cells without causing any harm to the cells [33]. Recent research has also demonstrated that overexpression of SIRT4 can reduce podocyte injury and inhibit podocyte apoptosis under hyperglycemic conditions by increasing the mitochondrial membrane potential (MMP) and reducing ROS originating from the mitochondria in podocytes [34]. To our knowledge, no research has established a link between SIRT4 and ferroptosis. Indeed, given the involvement of other family proteins like SIRT1 [15], SIRT3 [16], and SIRT7 [35] in ferroptosis and the findings that SIRT4 has a protective effect in HPMECs exposed to CSE stress [18], it is highly conceivable that SIRT4 may also inhibit ferroptosis triggered by CSE in A549 cells.

In conclusion, our study has uncovered a hitherto undocumented link between plasmalogen biosynthesis enzymes and ferroptosis induced by CSE. Moreover, we demonstrated that the deacetylation of GNPAT by SIRT4 yields a significant protective effect against CSE-induced ferroptosis. These results shed new light on the underlying mechanisms of ferroptosis in response to CSE and suggest that targeting the deacetylation of GNPAT via SIRT4 may be a promising avenue for investigating COPD.

### Electronic supplementary material

Below is the link to the electronic supplementary material.

Supplementary Material 1

## Data Availability

All data are available from the corresponding author with reasonable request.

## References

[1] Sheikh K, Coxson H, O,Parraga G (2016). This is what COPD looks like. Respirol (Carlton Vic).

[2] Halpin DMG, Criner GJ, Papi A, Singh D, Anzueto A, Martinez FJ, Agusti AA, Vogelmeier, Prevention of Chronic Obstructive Lung Disease. C F. Global Initiative for the Diagnosis, Management, and. The 2020 GOLD Science Committee Report on COVID-19 and Chronic Obstructive Pulmonary Disease. American journal of respiratory and critical care medicine. 2021, 203 1, 24–36. 10.1164/rccm.202009-3533SO PMC7781116 33146552

[3] Scanlon PD, Connett JE, Waller LA, Altose MD, Bailey WC, Buist AS, Tashkin D, P,Lung Health Study Research G (2000). Smoking cessation and lung function in mild-to-moderate Chronic Obstructive Pulmonary Disease. The Lung Health Study American Journal of Respiratory and Critical care Medicine.

[4] Hirschhorn T, Stockwell BR (2019). The development of the concept of ferroptosis. Free Radic Biol Med.

[5] Stockwell BR, Friedmann Angeli JP, Bayir H, Bush AI, Conrad M, Dixon SJ, Fulda S, Gascon S, Hatzios SK, Kagan VE, Noel K, Jiang X, Linkermann A, Murphy ME, Overholtzer M, Oyagi A, Pagnussat GC, Park J, Ran Q, Rosenfeld CS, Salnikow K, Tang D, Torti FM, Torti SV, Toyokuni S, Woerpel K (2017). A,Zhang D D. Ferroptosis: a regulated cell death Nexus linking metabolism, Redox Biology, and Disease. Cell.

[6] Yang WS, SriRamaratnam R, Welsch ME, Shimada K, Skouta R, Viswanathan VS, Cheah JH, Clemons PA, Shamji AF, Clish CB, Brown LM, Girotti AW, Cornish VW, Schreiber SL (2014). Stockwell B R. Regulation of ferroptotic cancer cell death by GPX4. Cell.

[7] Gaschler MM, Andia AA, Liu H, Csuka JM, Hurlocker B, Vaiana CA, Heindel DW, Zuckerman DS, Bos PH, Reznik E, Ye LF, Tyurina YY, Lin AJ, Shchepinov MS, Chan AY, Peguero-Pereira E, Fomich MA, Daniels JD, Bekish AV, Shmanai VV, Kagan VE, Mahal LK, Woerpel K (2018). A,Stockwell B R. FINO(2) initiates ferroptosis through GPX4 inactivation and iron oxidation. Nat Chem Biol.

[8] Homma T, Kobayashi S, Fujii J (2020). Cysteine preservation confers resistance to glutathione-depleted cells against ferroptosis via CDGSH iron sulphur domain-containing proteins (CISDs). Free Radic Res.

[9] Yoshida M, Minagawa S, Araya J, Sakamoto T, Hara H, Tsubouchi K, Hosaka Y, Ichikawa A, Saito N, Kadota T, Sato N, Kurita Y, Kobayashi K, Ito S, Utsumi H, Wakui H, Numata T, Kaneko Y, Mori S, Asano H, Yamashita M, Odaka M, Morikawa T, Nakayama K, Iwamoto T, Imai H (2019). Kuwano K. Involvement of cigarette smoke-induced epithelial cell ferroptosis in COPD pathogenesis. Nat Commun.

[10] Liu X, Ma Y, Luo L, Zong D, Li H, Zeng Z, Cui Y, Meng W, Chen Y (2022). Dihydroquercetin suppresses cigarette smoke induced ferroptosis in the pathogenesis of Chronic Obstructive Pulmonary Disease by activating Nrf2-mediated pathway. Phytomedicine: Int J Phytotherapy Phytopharmacology.

[11] Lian N, Zhang Q, Chen J, Chen M, Huang J, Lin Q (2021). The role of ferroptosis in Bronchoalveolar Epithelial Cell Injury Induced by cigarette smoke extract. Front Physiol.

[12] Braverman NE, Moser AB (2012). Functions of plasmalogen lipids in health and Disease. Biochim Biophys Acta.

[13] Honsho M, Fujiki Y (2017). Plasmalogen homeostasis - regulation of plasmalogen biosynthesis and its physiological consequence in mammals. FEBS Lett.

[14] Kong X, Guan J, Li J, Wei J, Wang R (2017). P66(Shc)-SIRT1 regulation of oxidative stress protects against cardio-cerebral vascular Disease. Mol Neurobiol.

[15] Chen H, Lin X, Yi X, Liu X, Yu R, Fan W, Ling Y, Liu Y, Xie W (2022). SIRT1-mediated p53 deacetylation inhibits ferroptosis and alleviates heat stress-induced lung epithelial cells injury. Int J Hyperthermia: Official J Eur Soc Hyperthermic Oncol North Am Hyperth Group.

[16] Han D, Jiang L, Gu X, Huang S, Pang J, Wu Y, Yin J, Wang J (2020). SIRT3 deficiency is resistant to autophagy-dependent ferroptosis by inhibiting the AMPK/mTOR pathway and promoting GPX4 levels. J Cell Physiol.

[17] Cai S, Fu S, Zhang W, Yuan X, Cheng Y, Fang J (2021). SIRT6 silencing overcomes resistance to sorafenib by promoting ferroptosis in gastric cancer. Biochem Biophys Res Commun.

[18] Chen Y, Wang H, Luo G, Dai X (2014). SIRT4 inhibits cigarette smoke extracts-induced mononuclear cell adhesion to human pulmonary microvascular endothelial cells via regulating NF-kappaB activity. Toxicol Lett.

[19] Gu L, Zhu Y, Lin X, Tan X, Lu B, Li Y (2020). Stabilization of FASN by ACAT1-mediated GNPAT acetylation promotes lipid metabolism and hepatocarcinogenesis. Oncogene.

[20] Ito A, Hashimoto M, Tanihata J, Matsubayashi S, Sasaki R, Fujimoto S, Kawamoto H, Hosaka Y, Ichikawa A, Kadota T, Fujita Y, Takekoshi D, Ito S, Minagawa S, Numata T, Hara H, Matsuoka T, Udaka J, Araya J, Saito M, Kuwano K (2022). Involvement of parkin-mediated mitophagy in the pathogenesis of chronic obstructive pulmonary disease-related Sarcopenia. J Cachexia Sarcopenia Muscle.

[21] Wang L, Pelgrim CE, Peralta Marzal LN, Korver S, van Ark I, Leusink-Muis T, van Helvoort A, Keshavarzian A, Kraneveld AD, Garssen J, Henricks PAJ, Folkerts G (2022). Braber S. changes in intestinal homeostasis and immunity in a cigarette smoke- and LPS-induced murine model for COPD: the lung-gut axis. Am J Physiol Lung Cell Mol Physiol.

[22] Zhuo R, Rong P, Wang J, Parvin R, Deng Y. The potential role of bioactive plasmalogens in lung surfactant. Front cell Dev Biology. 2021, 9 618102. 10.3389/fcell.2021.618102 PMC7928286 33681198

[23] Lee H, Zhuang L, Gan B (2021). Ether phospholipids govern ferroptosis. J Genet Genomics = Yi Chuan Xue bao.

[24] Sun X, Feng X, Zheng D, Li A, Li C, Li S, Zhao Z (2019). Ergosterol attenuates cigarette smoke extract-induced COPD by modulating inflammation, oxidative stress and apoptosis in vitro and in vivo. Clin Sci.

[25] Cooper JR, Abdullatif MB, Burnett EC, Kempsell KE, Conforti F, Tolley H, Collins JE, Davies DE. Long Term Culture of the A549 Cancer Cell Line promotes multilamellar body formation and differentiation towards an alveolar type II pneumocyte phenotype. PLoS ONE. 2016, 11 10, e0164438. 10.1371/journal.pone.0164438 PMC5085087 27792742

[26] Li J, Liu J, Yue W, Xu K, Cai W, Cui F, Li Z, Wang W, He J (2020). Andrographolide attenuates epithelial-mesenchymal transition induced by TGF-beta1 in alveolar epithelial cells. J Cell Mol Med.

[27] Su X, Chen J, Lin X, Chen X, Zhu Z, Wu W, Lin H, Wang J, Ye X (2021). Zeng Y. FERMT3 mediates cigarette smoke-induced epithelial-mesenchymal transition through Wnt/beta-catenin signaling. Respir Res.

[28] Xu W, Yan J, Chen S, Ocak U, Shao A, Zhang J (2020). Peroxisomal Dysfunction contributes to White Matter Injury following subarachnoid Hemorrhage in rats via thioredoxin-interacting protein-dependent manner. Front cell Dev Biology.

[29] Qin Y, Deng W, Ekmekcioglu S, Grimm EA (2013). Identification of unique sensitizing targets for anti-inflammatory CDDO-Me in metastatic Melanoma by a large-scale synthetic lethal RNAi screening. Pigment cell & Melanoma Research.

[30] Elkhwanky MS, Hakkola J (2018). Extranuclear sirtuins and metabolic stress. Antioxid Redox Signal.

[31] Wu T, Liu YH, Fu YC, Liu X, M,Zhou XH (2014). Direct evidence of sirtuin downregulation in the liver of non-alcoholic fatty Liver Disease patients. Ann Clin Lab Sci.

[32] Osborne B, Bentley NL, Montgomery MK, Turner N (2016). The role of mitochondrial sirtuins in health and Disease. Free Radic Biol Med.

[33] Park ES, Kang JC, Jang YC, Park JS, Jang SY, Kim DE, Kim B, Shin HS (2014). Cardioprotective effects of rhamnetin in H9c2 cardiomyoblast cells under H(2)O(2)-induced apoptosis. J Ethnopharmacol.

[34] Shi JX, Wang QJ, Li H, Huang Q (2017). SIRT4 overexpression protects against diabetic Nephropathy by inhibiting podocyte apoptosis. Experimental and Therapeutic Medicine.

[35] Li XT, Song JW, Zhang ZZ, Zhang MW, Liang LR, Miao R, Liu Y, Chen YH, Liu X, Y,Zhong JC. Sirtuin 7 mitigates renal ferroptosis, fibrosis and injury in hypertensive mice by facilitating the KLF15/Nrf2 signaling. Volume 193. Free radical biology & medicine; 2022. pp. 459–73. Pt 1. 10.1016/j.freeradbiomed.2022.10.320 36334846

