# Supplementary material for: CSE triggers ferroptosis via SIRT4-mediated GNPAT deacetylation in the pathogenesis of COPD
Source: Respir Res. 2023 Dec 1;24:301. doi: 10.1186/s12931-023-02613-0 (PMC10691148; doi:10.1186/s12931-023-02613-0)
Supplement: Supplementary file 1 — Supplementary Material 1 [file 12931_2023_2613_MOESM1_ESM.pdf]

Figure 1

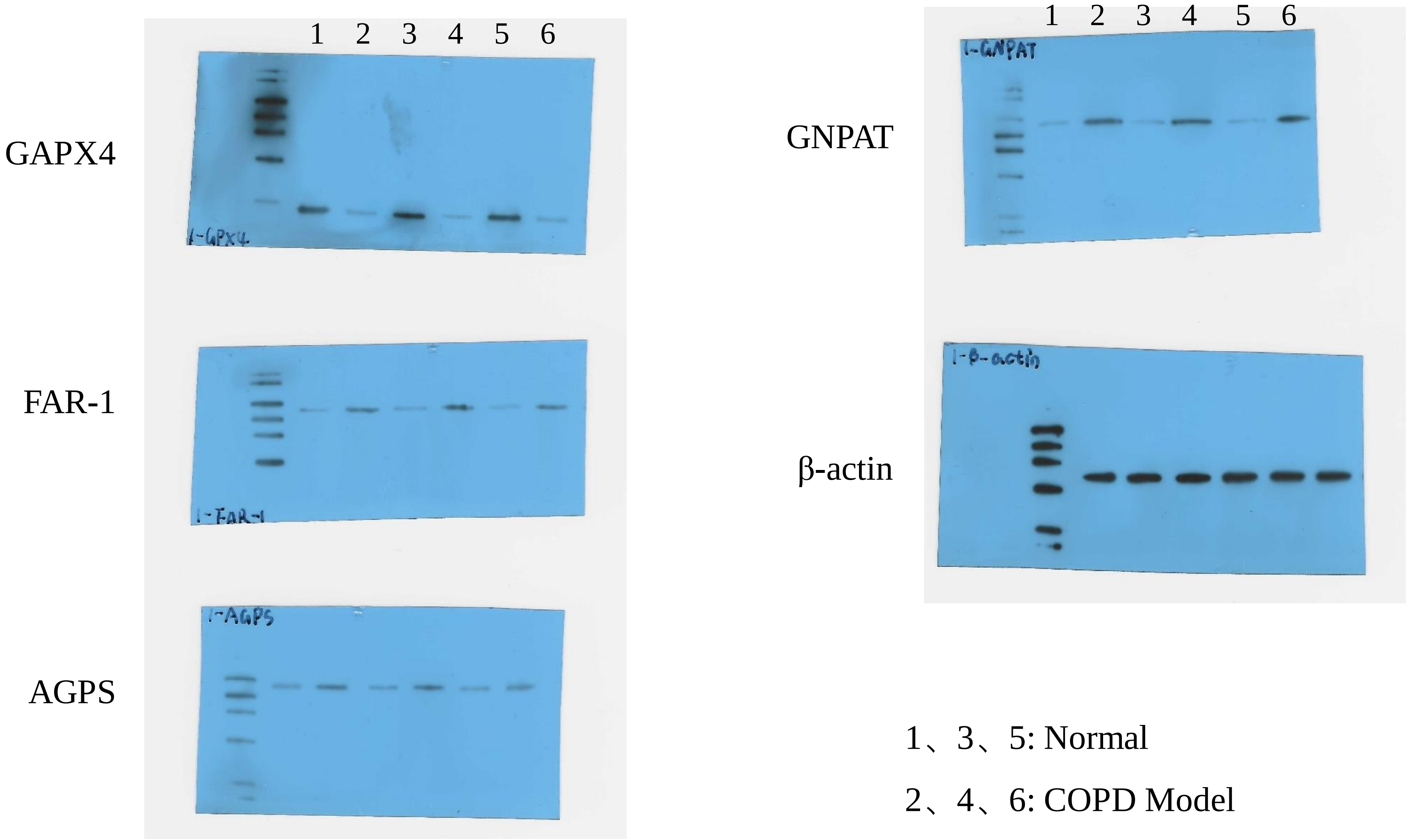
1 2 3 4 5 6
GAPX4
1-GPX4
FAR-1
1-FAR-1
AGPS
1-AGPS
1 2 3 4 5 6
GNPAT
1-GNPAT
β-actin
1-β-actin
1、3、5: Normal
2、4、6: COPD Model

Figure 2

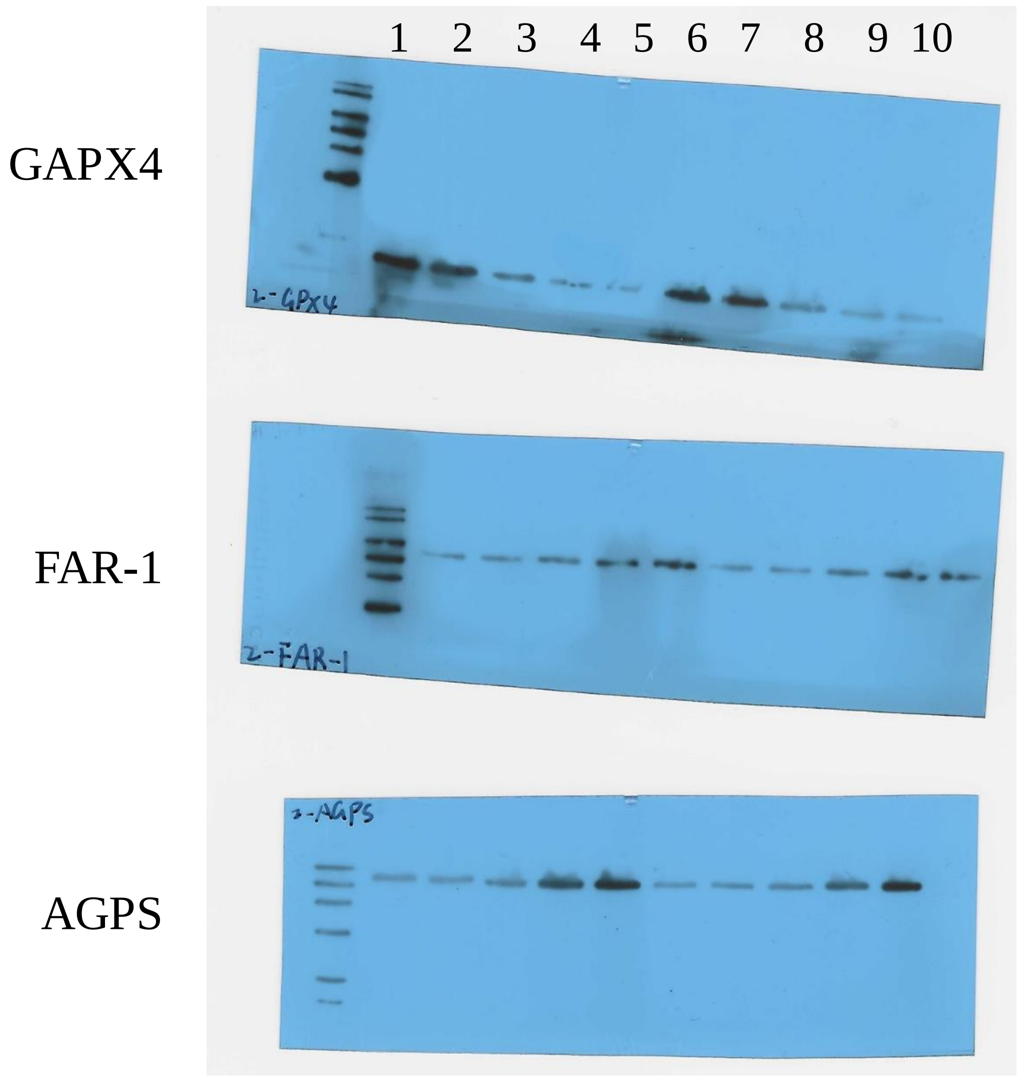

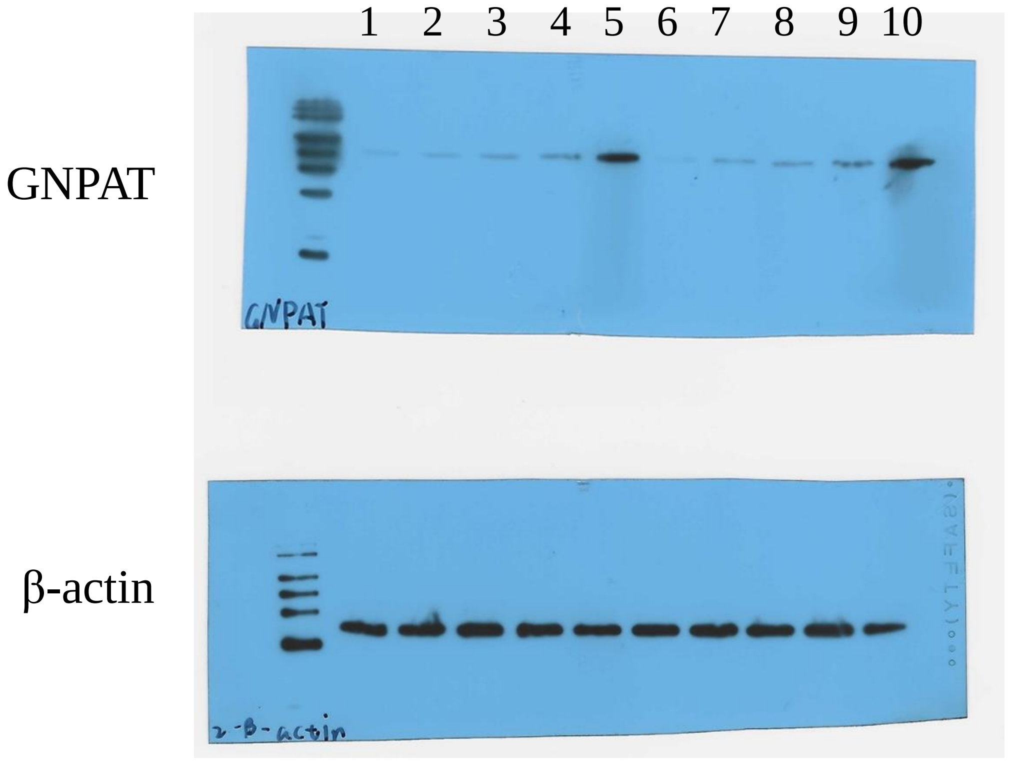

1、6: Blank

2、7: 0.1%CSE

3、8 : 0.5%CSE

4、9 : 2%CSE

5、10 : 5%CSE

Figure 4

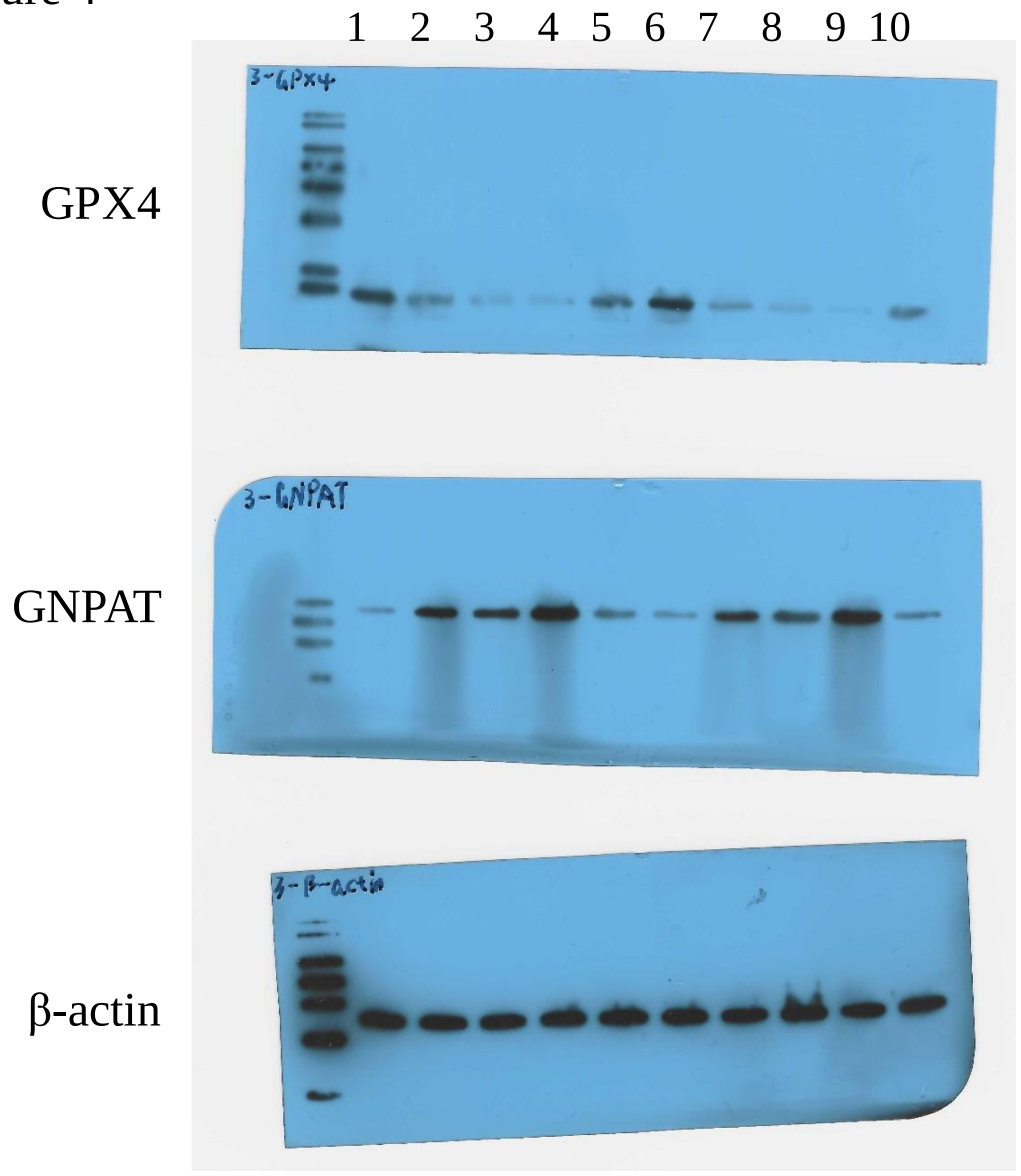

1、6: Blank

2、7: 5%CSE

3、8 : 5%CSE+DMSO

4、9 : 5%CSE+Erastin

5、10 : 5%CSE+Fer-1

Figure 5

GNPAT

β-actin

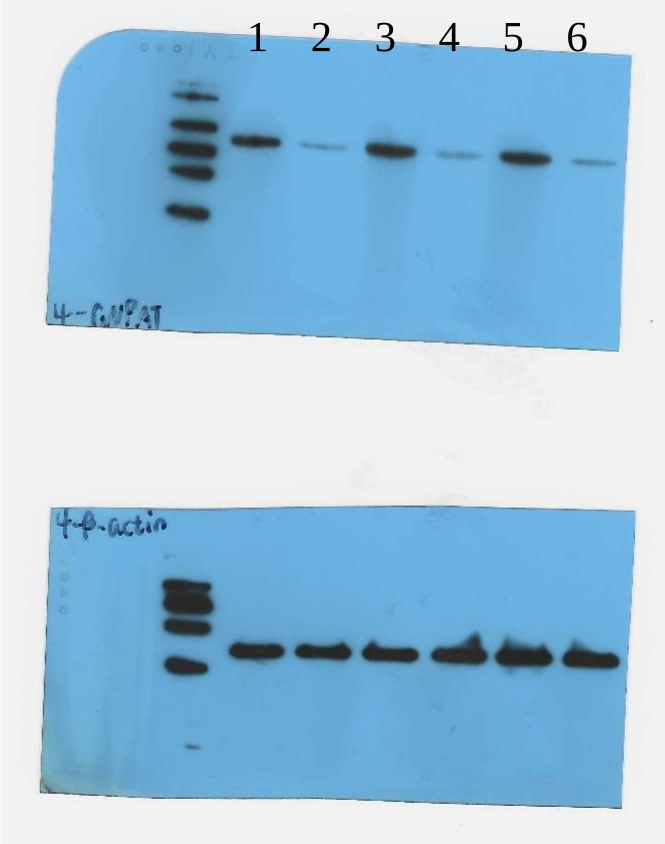

1、3、5: sh-NC

2、4、6: sh-GNPAT

Figure 5

SIRT4

β-actin

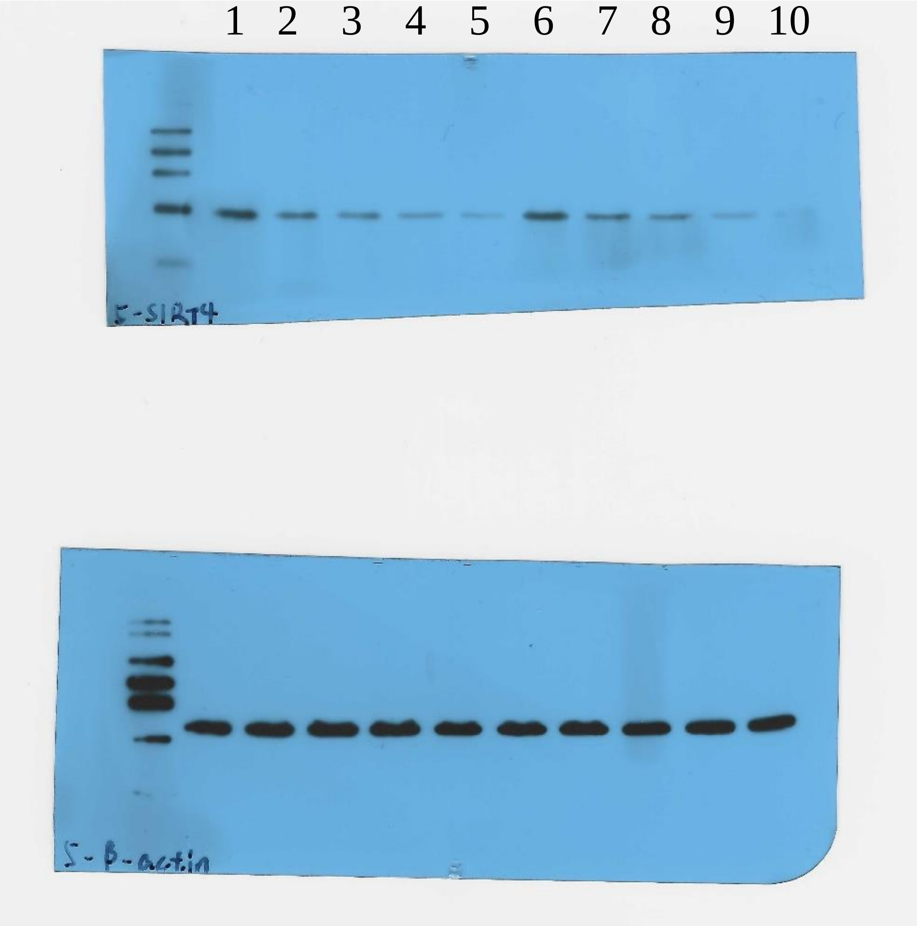

Acetyl-
lysine

GNPAT

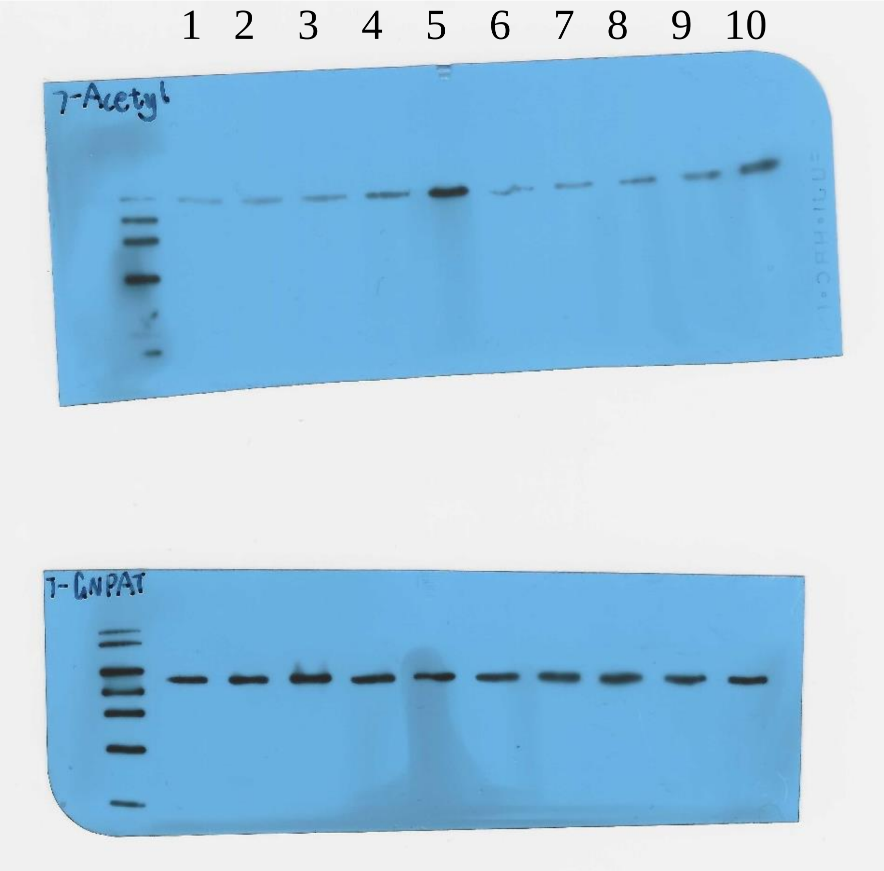

1、6: Blank

2、7: 0.1%CSE

3、8 : 0.5%CSE

4、9 : 2%CSE

5、10 : 5%CSE

Figure 6

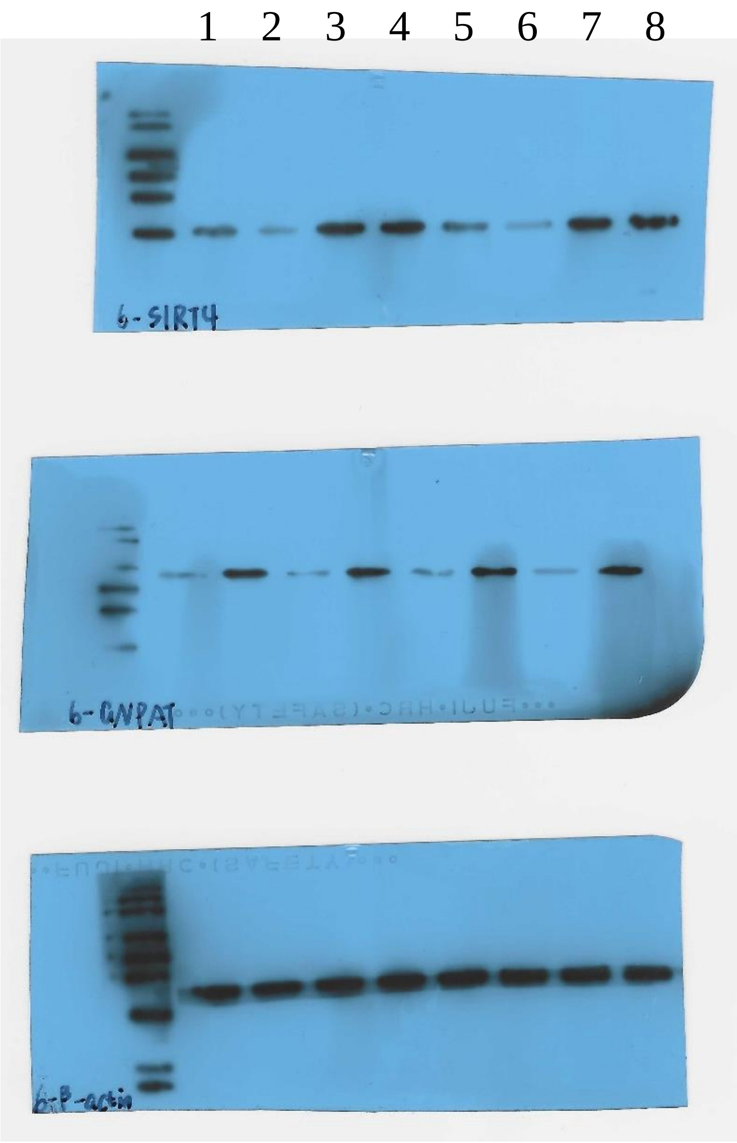

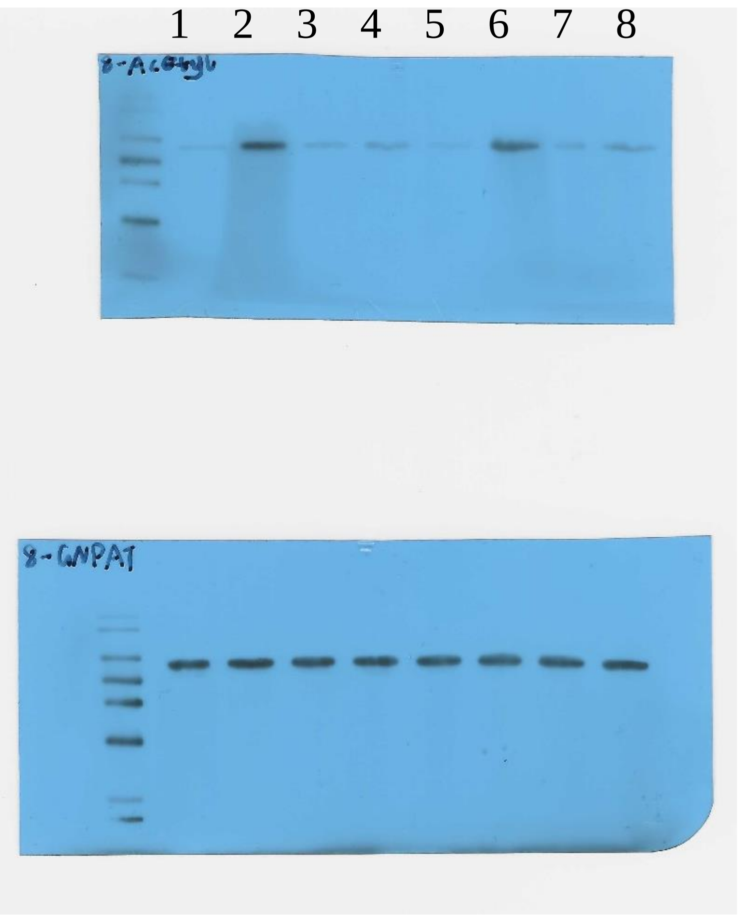

1、5: Blank

2、6: 5%CSE+vector

3、7 : 5%CSE+SIRT4

4、8 : 5%CSE+SIRT4+GNPAT
